# Bone mineral density and osteoporosis risk in young adults with atopic dermatitis

**DOI:** 10.1038/s41598-021-03630-z

**Published:** 2021-12-20

**Authors:** Sooyoung Kim, Jimi Choi, Moon Kyun Cho, Nam Hoon Kim, Sin Gon Kim, Kyeong Jin Kim

**Affiliations:** 1 Department of Dermatology, Soonchunhyang University Hospital, Seoul, Republic of Korea; 2 Division of Endocrinology and Metabolism, Department of Internal Medicine, Korea University Anam Hospital, Korea University College of Medicine, 73 Inchon-ro, Seongbuk-gu, Seoul, 02841 Republic of Korea

**Keywords:** Endocrinology, Skin diseases

## Abstract

Atopic dermatitis (AD) has been increasing worldwide over the past few decades. AD has been reported to be associated with an increased risk of osteoporosis and fractures in adult AD patients. The aim of this study was to investigate the bone mineral density (BMD) to evaluate osteoporosis risk in young adults with AD by sex. This was a case–control cohort study using a national dataset from the Korea National Health and Nutrition Examination Survey 2007–2009. We included young adult AD patients (men aged 19 ≤ and < 50 years, premenopausal women aged 19 ≤ and < 50 years) and 1:5 propensity score weighting controls by age, sex, body mass index (BMI), vitamin D level, and alcohol/smoking status. BMD was measured by double energy X-ray absorptiometry at the lumbar spine, femur neck, and total femur. The prevalence of low BMD, defined by a Z-score ≤  − 2.0, was compared between AD and without AD. We analyzed 311 (weighted n = 817,014) AD patients and 8,972 (weighted n = 20,880,643) controls. BMD at the lumbar spine was significantly lower in the male AD group than in the male control group (mean ± SE, 0.954 ± 0.016 vs. 0.989 ± 0.002, *P* = 0.03). The prevalence of low BMD (Z-score) did not significantly differ between AD and non-AD subjects in both men (3.8% *vs*. 2.7%, *P* = 0.56) and women (6.4% *vs*. 3.3%, *P* = 0.40). Among AD patients, early age at diagnosis of AD, longer duration of AD, lower BMI, rural residence (for men), less education, low vitamin D level, late menarche, and more pregnancies (for women) were associated with low BMD. In conclusion, low BMD did not occur more frequently in young adults with AD than in non-AD controls. However, early-onset/longer AD duration and lower BMI were associated with low BMD among young adult patients with AD.

## Introduction

Atopic dermatitis (AD) is one of the most common inflammatory skin diseases affecting 15–30% of children and 2–10% of adults^1^. The AD prevalence has been increasing worldwide in recent decades. As AD is a chronic inflammatory disease that usually begins in early childhood or adolescence, various comorbidities have been associated with AD. Bone health problems in patients with AD have increasingly received attention because AD is associated with risk factors for osteopenia and osteoporosis, including topical/systemic corticosteroid usage, less physical activity, malnutrition due to food restriction, and chronic inflammatory disease course^2–4^. Haeck et al. reported that low bone mineral density (BMD), defined as a Z-score of ≤  − 1, was prevalent in adults with moderate-to-severe AD^5^. Another study from the UK showed that patients with atopic eczema are associated with a 10% increased fracture risk compared with those without this condition^6^. However, existing studies have several limitations. First, most results included the elderly population. Since (1) older age is the major risk factor for osteoporosis and fracture, (2) hormonal changes in postmenopausal women have a great influence on bone health, (3) and the elderly population might have many comorbidities such as cardiovascular disease, diabetes, and chronic kidney diseases that may affect bone health, which cannot represent the bone health of the majority of young adult patients with AD. Second, multiple factors that affect bone health, such as sex, vitamin D level, alcohol consumption, smoking, and menopausal status, have not been fully applied in previous studies^1^. A few studies have evaluated the prevalence of low BMD and osteoporosis risk in young patients with AD, particularly for the accurate measurement of BMD using DXA. Thus, we aimed to investigate the BMD and Z-score using a large sample of young adults aged 19–49 years old with AD and non-AD controls with propensity score weighting for age, sex, body mass index (BMI), vitamin D level, and alcohol/smoking status using data from the Korean National Health and Nutrition Examination Survey (KNHANES) 2007–2009.

## Materials and methods

### Study population and design

The KNHANES is a cross-sectional nationwide survey conducted by the Korea Centers for Disease Control and Prevention (KCDC)^7^. It has been conducted periodically since 1998, and data were gathered through household interviews, standardized physical examinations, laboratory tests, and nutritional status assessments. The KNHANES IV, which only performed the BMD test, was conducted between 2008 and 2011, including more than 260,000 primary sampling units each year. After applying the stratified, multi-stage clustered systemic sampling method, 19,841 of the 25,250 systemically selected individuals participated in this survey. We only included young adults aged 19 ≤ and < 50 years. Then, we excluded (1) those with incomplete information on the atopic disease, BMD results, BMI, and 25(OH)D level; or (2) postmenopausal women for any reason. We defined AD if a subject answered positively to a questionnaire “Have you ever been diagnosed with AD by a physician?”. Atopy treatment was defined if a subject answered positively to the question, “Do you currently receive any kind of treatment for atopic dermatitis?”. The comorbidities were defined based on the response to the question regarding whether a subject had ever been diagnosed with the disease (i.e., hypertension, diabetes, depression, asthma, etc.) by a physician. Sickness in 1 month was defined if a subject answered positively to the question “Have you ever suffered from any kind of diseases or injuries during the recent 1 month?”. Absence in 1 month was defined if a subject answered positively to the question “Have you ever been absent from work or school due to any kind of disease or injury in the past 1 month?”. All survey protocols were approved by the Institutional Review Board of the KCDC and we confirmed that all methods were performed in accordance with the relevant guidelines and regulation. All participants signed an informed consent form before the start of the survey.

### Bone densitometry and definition

BMD at the lumbar spine, femoral neck, and total hip was measured using dual-energy X-ray absorptiometry (DXA, QDR4500A System; Hologic Inc., Waltham, MA, USA). BMD was expressed as the total bone mineral content (g) divided by the area (cm^2^). We calculated the Z-score of the lumbar spine, femoral neck, and total hip using the age- and sex-specific mean BMD (g/cm^2^): (patient BMD–mean BMD of reference group)/(standard deviation [SD] of reference group). The SDs for the complex survey design were calculated using the Taylor series linearization method^8^. The weighted mean and SD of the reference BMD for age and sex used to calculate the Z-score are described in Supplementary Table 1. According to the World Health Organization diagnostic classification, we defined low bone mass based on the expected range for their age group as a Z-score of ≤  − 2.0 and normal as a Z-score of >  − 2.0^9^.

### Statistical analyses

To obtain appropriate nationally representative estimates, all analyses in this study were performed considering the complex survey design applied to the KNHANES. The propensity score weighting method was used to balance the variables between patients with and without AD. Individual propensity scores were calculated using multiple logistic regression models, including the presence of atopy as the dependent variable and age, sex, BMI, vitamin D level, alcohol consumption, smoking, and complex survey sampling weight as covariates. The final weight was obtained by multiplying the sampling weight by the propensity score weight^10^. Continuous data were presented as the weighted mean and standard error of the mean for normally distributed variables and as medians and interquartile ranges (IQRs) for non-normally distributed variables. Categorical data are expressed as weighted frequencies and percentages. To compare the characteristics between patients with AD and those without AD, we performed a weighted t-test for continuous variables and the Rao-Scott chi-square test for categorical variables. Finally, multiple logistic regression models considering the complex survey design were used to investigate the risk factors for the low BMD group according to sex. In this model, the low BMD group was included as a binary outcome variable, and age, atopic dermatitis, body mass index, vitamin D deficiency, education, drinking, and age at menarche (female only) were included as dependent variables. The results of logistic regression analysis are presented as odds ratios (ORs) and corresponding 95% confidence intervals (CIs). All reported *P*-values were two-sided, and statistical significance was set at *P* < 0.05. All statistical analyses were performed using the SAS software (version 9.4; SAS Institute Inc., Cary, NC, USA).

## Results

### Participants’ clinical characteristics

This study included 311 (weighted n = 817,014) patients with AD and 8,972 (weighted n = 20,880,643) without AD. The incidence of AD in the young adult population was 3.6% in men and 4.0% in women. The median ages at AD diagnosis were 18.7 years (IQR: 8.6–31.3 years) and 17.0 years (IQR 8.4–26.2 years) in male and female patients with AD, with median durations of AD 9.8 years (IQR: 4.7–19.3 years) and 10.8 years (IQR: 5.5–19.1 years), respectively. Meanwhile, 29.7% of male and 20.5% of female AD patients received atopy treatment at the time of the survey. Among female patients, the median age at menarche was not significantly different between patients with AD and the control group. Higher family income was more frequently reported in the AD group than in the control group among female patients. Among female patients, no significant difference was observed in the history of oral contraceptive use between patients with AD and the control group. The experience of pregnancy was significantly lower in the AD group than in the non-AD control group (59.5 *vs*. 70.9%; *P* = 0.012). The prevalence of asthma was significantly higher in male with AD (10.4% vs. 2.0%; *P* = 0.003) and in female with AD (5.5 *vs*. 2.0%; *P* = 0.022) than in the non-AD groups of either sex. The prevalence of hypertension, diabetes mellitus, chronic kidney disease, dyslipidemia, osteoarthritis or rheumatoid arthritis, and depression was not significantly different in patients with AD compared to the control group in both sexes (*P* > 0.05) (Table 1).

**Table 1:** Baseline characteristics after propensity score weighting of atopic dermatitis and non-atopic dermatitis group according to the sex.

	Male (n = 11,585,713)		*P*	Female (n = 10,111,944)		*P*
	Non-AD	AD		Non-AD	AD	
	11,170,803 (96.4)	414,910 (3.6)		9,709,840 (96.0)	402,104 (4.0)	
Age, years (median, IQR)	34.6 (26.9–41.5)	35.3 (28.1–43.1)	0.388	34.5 (26.8–40.9)	34.2 (26.2–41.0)	0.833
BMI (mean ± SE), kg/m^2^	24.1 (0.07)	24.0 (0.32)	0.565	22.5 (0.07)	22.4 (0.31)	0.749
Age at diagnosis of atopic dermatitis, years (median, IQR)		18.7 (8.6–31.3)			17.0 (8.4–26.2)	
Duration of atopic dermatitis, years (median, IQR)		9.8 (4.7–19.3)			10.8 (5.5–19.1)	
Atopy treatment, n (%)		112,587 (29.7)			82,610 (20.5)	
**Education**			0.728			0.140
< High school	760,972 (6.8)	35,450 (8.5)		875,369 (9.0)	33,600 (8.4)	
High school	5,429,479 (48.6)	179,275 (43.2)		4,851,447 (50.0)	162,134 (40.3)	
College or more	4,975,112 (44.6)	200,185 (48.2)		3,983,024 (41.0)	206,369 (51.3)	
**Monthly family income (quartile), n (%)**			0.969			**0.019**
Lowest	2,887,602 (26.1)	106,009 (25.7)		2,565,049 (26.7)	114,897 (28.6)	
Lower middle	2,805,126 (25.4)	104,717 (25.3)		2,499,467 (26.0)	62,343 (15.5)	
Higher middle	2,737,561 (24.8)	112,551 (27.2)		2,400,287 (25.0)	91,443 (22.7)	
Highest	2,612,419 (23.7)	89,963 (21.8)		2,135,211 (22.2)	133,421 (33.2)	
**Residence area, n (%)**			0.087			0.993
Urban	9,451,039 (84.6)	377,968 (91.1)		8,361,707 (86.1)	346,158 (86.1)	
Rural	1,719,764 (15.4)	36,942 (8.9)		1,348,133 (13.9)	55,946 (13.9)	
**Alcohol, n (%)**			0.391			0.579
Never drinker	345,094 (3.1)	22,013 (5.3)		712,623 (7.3)	35,796 (8.9)	
Ex-/current drinker	10,825,708 (96.9)	392,897 (94.7)		8,997,217 (92.7)	366,308 (91.1)	
**Smoking, n (%)**			0.773			0.752
Never smoker	2,387,107 (21.4)	93,719 (22.6)		8,134,305 (83.8)	332,559 (82.7)	
Ex-/current smoker	8,783,696 (78.6)	321,191 (77.4)		1,575,535 (16.2)	69,545 (17.3)	
**Moderate exercise, n (%)**			0.239			0.247
Yes	1,451,455 (13.0)	38,957 (9.4)		1,057,025 (10.9)	57,487 (14.3)	
No	9,717,518 (87.0)	375,953 (90.6)		8642,770 (89.1)	344,617 (85.7)	
Vitamin D level, ng/dL (median, IQR)	17.2 (13.5–22.1)	17.0 (13.8–22.1)	0.716	14.8 (11.7–18.7)	15.4 (11.9–18.8)	0.816
Age at menarche, years (median, IQR)	–	–		13.1 (12.1–14.1)	12.9 (11.7–14.4)	0.842
OCP use, n (%)	–	–		1,106,502 (12.1)	38,651 (10.0)	0.434
Pregnancy experience, n (%)				6,488,925 (70.9)	229,307(59.5)	**0.012**
Number of pregnancies, median (IQR)	–	–		1.5 (0–2.8)	1.1 (0–2.5)	0.129
Hypertension, n (%)	707,637 (6.3)	31,232 (7.5)	0.641	254,012 (2.6)	5776 (1.4)	0.428
Diabetes mellitus, n (%)	200,698 (1.8)	5528 (1.3)	0.764	142,598 (1.5)	1389 (0.3)	0.115
Chronic kidney disease, n (%)	22,335 (0.2)	0 (0)	–	18,321 (0.2)	0 (0)	–
Dyslipidemia, n (%)	476,054 (4.3)	11,791 (2.8)	0.556	186,617 (1.9)	8750 (2.2)	0.835
OA or RA, n (%)	168,561 (1.5)	6300 (1.5)	0.994	282,554 (2.9)	15,998 (4.0)	0.556
Asthma, n (%)	226,949 (2.0)	43,281 (10.4)	**0.003**	196,188 (2.0)	22,043 (5.5)	**0.022**
Depression, n (%)	124,095 (1.1)	2570 (0.6)	0.558	349,371 (3.6)	16,025 (4.0)	0.838
Absence in 1 month, n (%)	362,195 (3.4)	23,418 (6.1)	0.404	393,547 (4.9)	29,706 (8.0)	0.106
Sickness in 1 month, n (%)	538,454 (4.8)	37,095 (8.9)	0.373	836,511 (8.6)	66,796 (16.6)	**0.003**

*AD* atopic dermatitis, *BMI* body mass index, *IQR* interquartile range, *OCP* oral contraceptive pill, *SE* standard error, *OA* osteoarthritis, *RA* rheumatoid arthritis.

Significant values are in bold.

### Bone mineral density and Z-score between the AD and control groups

BMD at the lumbar spine was lower in male with AD than in the control group (mean ± SE, 0.954 ± 0.016 vs. 0.989 ± 0.002; *P* = 0.03). BMD in the total femur was lower in AD patients than in the control group (0.977 ± 0.012 vs. 0.999 ± 0.002; *P* = 0.08), with borderline significance. For female AD patients, BMDs at the lumbar spine, femur neck, and total femur were not significantly different between the AD and control groups (Table 2). The prevalence rates of low BMD, defined by the lowest Z-score ≤  − 2.0 in the three measured sites (lumbar spine, total femur, and femoral neck), were not significantly different in the AD group compared with the control group in both male (3.8% vs. 2.7%; *P* = 0.56) and female (6.4% vs. 3.3%; *P* = 0.40) (Table 3).

**Table 2:** Comparisons of BMD at the lumbar spine, femur neck, and total hip between atopic dermatitis and non-atopic dermatitis patients.

	Lumbar spine BMD (g/cm^2^)	*P*	Femur neck BMD (g/cm^2^)	*P*	Total hip BMD (g/cm^2^)	*P*
**Male**		**0.030**		0.102		0.080
Non-AD	0.989 ± 0.002		0.863 ± 0.002		0.999 ± 0.002	
AD	0.954 ± 0.016		0.843 ± 0.011		0.977 ± 0.012	
**Female**		0.744		0.936		0.411
Non-AD	0.985 ± 0.002		0.765 ± 0.002		0.898 ± 0.002	
AD	0.979 ± 0.016		0.766 ± 0.010		0.890 ± 0.010	
*P* for interaction*		0.213		0.215		0.439

*BMD* bone mineral density, *AD* atopic dermatitis.

Data are expressed as mean ± standard error.

*P for the interaction between sex and atopy.

Significant values are in bold.

**Table 3:** Comparisons of Z-score at the lumbar spine, femur neck, and total hip between atopic and non-atopic patients.

	Male (N = 1,1585,713)				*P*	Female (N = 10,111,944)				*P*
	Non-AD		AD			Non-AD		AD		
**Lumbar spine**					0.139					0.213
Z-score > − 2.0	10,777,887	(98.6)	398,165	(96.7)		9,312,514	(98.3)	377,645	(94.6)	
Z-score ≤ − 2.0	148,098	(1.4)	13,580	(3.3)		161,638	(1.7)	21,550	(5.4)	
**Femoral neck**					0.830					0.406
Z-score > − 2.0	11,026,396	(99.2)	404,088	(99.3)		9,429,111	(98.7)	392,829	(99.4)	
Z-score ≤ − 2.0	88,805	(0.8)	2,839	(0.7)		126,379	(1.3)	2,316	(0.6)	
**Total hip**					0.834					0.214
Z-score > − 2.0	10,927,793	(98.3)	401,067	(98.6)		9,394,880	(98.3)	393,091	(99.5)	
Z-score ≤ − 2.0	187,408	(1.7)	5860	(1.4)		160,610	(1.7)	2,053	(0.5)	
**Normal/Low**					0.555					0.395
Normal group	10,864,302	(97.3)	399,326	(96.2)		9,392,180	(96.7)	376,185	(93.6)	
Low Z-score group*	306,501	(2.7)	15,584	(3.8)		317,660	(3.3)	25,919	(6.4)	

*AD* atopic dermatitis.

*Z-score ≤  − 2.0 in at least one of three sites.

### Risk factors for low BMD among patients with AD stratified by sex

We compared the clinical characteristics of the normal and low BMD groups among patients with AD stratified by sex. Among male patients with AD, the low BMD group was diagnosed with AD at a younger age, lower BMI, and frequently residing in rural areas compared with the normal BMD group. In female patients with AD, the low BMD group was diagnosed with AD at an earlier age and longer AD duration. The low BMD group had lower BMI, less education, lower vitamin D level, later age at menarche, and higher number of pregnancies compared with the normal BMD group (Table 4).

**Table 4:** Risk factors for low bone mineral density among patients with atopic dermatitis by sex.

	Male AD patients (n = 414,910)		*P*	Female AD patients (n = 402,104)		*P*
	Normal BMD	Low BMD		Normal BMD	Low BMD	
Total cases, n (%)	399,326 (96.2)	15,584 (3.8)		376,185 (93.6)	25,919 (6.4)	
Age at diagnosis of AD, years (median, IQR)	22.9 (11.3–34.8)	19.6 (8.7–36.4)	** < 0.001**	21.8 (11.0–29.6)	14.0 (12.5–15.5)	** < 0.001**
Duration of AD, years (median, IQR)	9.4 (4.6–18.9)	3 (3–19.4)	0.284	10.4 (5.2–19.1)	31.8 (31.2–32.4)	** < 0.001**
Atopy treatment, n (%)	110,595 (30.4)	1,992 (12.8)	0.335	64,874 (18.8)	0 (0)	–
BMI (mean ± **SE**), kg/m^2^	24.0 (0.28)	23.0 (1.19)	**0.013**	22.4 ± 0.32	21.4 ± 0.74	** < 0.001**
**Education**			0.748			**0.009**
≤ High school	205,387 (51.4)	9,338 (59.9)		172,131 (45.8)	23,603 (91.1)	
College or more	193,940 (48.6)	6,245 (40.1)		204,054 (54.2)	2316 (8.9)	
**Monthly family income (quartile), n (%)**			0.331			–
Lowest	98,277 (24.7)	7,732 (49.6)		112,844 (30.0)	2,053 (7.9)	
Lower middle	103,881 (26.1)	835 (5.4)		62,343 (16.6)	0 (0)	
Higher middle or highest	195,498 (49.2)	7,016 (45.0)		200,998 (53.4)	23,866 (92.1)	
**Residence area, n (%)**			**0.018**			–
Urban	368,884 (92.4)	9,084 (58.3)		320,239 (85.1)	25,919 (100)	
Rural	30,442 (7.6)	6,499 (41.7)		55,946 (14.9)	0 (0)	
**Alcohol, n (%)**			0.735			–
Never drinker	20,780 (5.2)	1,233 (7.9)		35,796 (9.5)	0 (0)	
Ex-/current drinker	378,546 (94.8)	14,351 (92.1)		340,389 (90.5)	25,919 (100)	
**Smoking, n (%)**			0.295			–
Never smoker	92,113 (23.1)	1,606 (10.3)		69,545 (18.5)	0 (0)	
Ex-/current smoker	307,214 (76.9)	13,978 (89.7)		306,640 (81.5)	25,919 (100)	
**Moderate exercise, n (%)**			0.870			0.600
Yes	37,724 (9.4)	1,233 (7.9)		55,434 (14.7)	2,053 (7.9)	
No	361,602 (90.6)	14,351 (92.1)		320,751 (85.3)	238,66 (92.1)	
Vitamin D level, ng/dL (median, IQR)	16.8 (13.5–21.3)	32.1 (26.0–33.7)	** < 0.001**	15.2 (11.9–18.9)	14.4 (12.9–15.9)	** < 0.001**
Age at menarche, years (median, IQR)	–	–	–	12.8 (11.6–14.0)	15.0 (13.5–16.5)	** < 0.001**
OCP use, n (%)	–	–	–	36,598 (10.2)	2,053 (7.9)	0.840
Pregnancy experience, n (%)	–	–	–	203,388 (56.5)	25,919 (100)	–
Number of pregnancies, (median, IQR)	–	–	–	0.8 (0–2.3)	2.2 (2.5–2.8)	** < 0.001**

*AD* atopic dermatitis, *BMI* body mass index, *OCP* oral contraceptive pill, *IQR* interquartile range, *SE* standard error.

*Low BMD was defined as a Z-score of ≤  − 2.

Significant values are in bold.

## Discussion

This retrospective weighted case–control study demonstrated that the prevalence of low bone mass (Z-score ≤  − 2.0) in the AD group was increased but not significantly different from that in the non-AD group in either sex. BMD at the lumbar spine was significantly lower in AD patients than in the control group, but there was no significant difference in the other two sites among men. There was no difference in BMD at any of the three sites among women. Among AD patients, early age at diagnosis of AD, longer duration of AD, lower BMI, rural living (for men), less educated, low vitamin D level, late menarche, and higher number of pregnancies (for women) were associated with low BMD.

In baseline characteristics after propensity score weighting, a lower number of pregnancies was reported among women with AD, although oral contraceptive use was comparable. A higher frequency of single or never-married status of AD patients^11^, psychological distress, including depression, anxiety, and lower self-esteem of AD people^12,13^, may affect their partner relationships^14^, which explains the low pregnancy rates among AD patients. AD patients had an increased prevalence rates of asthma 10.4% for male and 5.5% for female AD patients compared to those of non-AD group which is consistent with the prevalence rates of physician-diagnosed asthma as 2.0% reported in the previous studies^11^. On the other hand, no difference was found in the prevalence of hypertension, diabetes, chronic kidney disease, osteoarthritis/ rheumatoid arthritis, or depression between the AD and non-AD groups. Considering that we matched vitamin D, as well as age, sex, drinking and smoking status those which have been known for their effects on these chronic diseases, the comorbidities of AD group in our study could be turned out not to be significantly different from non-AD group^15–18^. Women with AD were more likely to fall ill due to any kind of disease or injury within 1 month than non-AD women, although no significant difference was found within 1 month of absence from work or school in this study. According to a meta-analysis on the influence of AD on work life, AD affects sick leave, possibly affecting job choice and leading to change or loss of job^19^.

We found a significant decrease in BMD in the lumbar spine of male patients with AD. A previous report also found significant decrease only in the lumbar spine of AD patients compared to non-AD controls but no difference between two groups in the BMD was found of other sites (femoral neck, trochanter, intertrochanteric crista, Ward’s triangle, and calcaneus)^2^. It has been suggested that in adults, approximately 25% of the trabecular bone is replaced every year, while 3% of the cortical bone does^2^. Considering the higher turnover rates of trabecular bones, glucocorticoid effects on the bone are more likely to be found in areas rich in trabecular bone, (i.e. vertebrae) such as lumbar region^2^. The decrease of BMD in lumbar spine could be considered as an early sign of BMD reduction in AD patients because the lumbar spine is more responsive to metabolic changes^20,21^. However, BMD change could be interpreted as reflecting a “real” change only if the change exceeded the random error of the DXA system. The random error or least significant change has been described to be between 3.3 and 4.7% for the total hip^22,23^. The decrease in BMD in AD patients was 3.5% in our study compared with that in non-AD controls, which is marginal; it appears to be not a relevant decrease.

The prevalence of low BMD was 3.8% in men and 6.7% in women with AD in our study, but these increases were not statistically significant compared to non-AD groups of either sex. There are several possible explanations for the lack of a significant association between AD and low BMD. First, AD is a type 2 inflammation and Th2 cytokines are recognized to have an osteoprotective effect, enhancing the anabolic effects of parathyroid hormone, and decreasing the receptor activator of nuclear factor-kB ligand/osteoprotegerin ratio, leading to inhibition of bone resorption^24^. Secondly, the treatment of atopic dermatitis could affect bone health less than expected. Oral corticosteroids are a well-recognized risk factor for osteoporosis; however, in a population study by Lowe et al.^6^, no significant association was found between oral steroid use and fracture risk, possibly because of the small number of participants who had been receiving long-term steroids as treatment for AD. In addition, as systemic steroids are recommended for acute flare of AD, long-term use of high doses of systemic steroids is rarely performed by dermatologists. There is little evidence to suggest that topical steroids, even potent topical steroids, are associated with osteoporosis^5,21,22,25^.

There are several reports that found no significant decrease in BMD in AD patients compared to non-AD controls in both adults and children. A study from Finland, including 28 adult AD patients, demonstrated that the BMDs of patients with AD did not differ from those of healthy controls^2^. There was no significant correlation between BMD and single risk factors, such as barrier function, duration of AD, and use of oral or topical glucocorticoids^2^. Only in selected group of AD patients, needing higher potency of topical glucocorticoids than hydrocortisone had lower values for lumbar BMD than non-AD controls^2^. Also, in children with moderate to severe AD, low BMD (defined as a Z score <  − 2) did not occur more frequently^25^. According to the study, low BMD was not associated with the use of topical corticosteroids in the previous 5 years in the children with moderate to severe AD^25^. In addition, BMD was decreased but statistically not significant in the children with moderate-to-severe AD who received systemic treatments (oral corticosteroids and/or cyclosporine) in the previous 5 years^25^.

In contrast, studies demonstrating low BMD rates, especially in patients with moderate to severe AD, have been increasingly reported. In a study by Haeck et al. including 125 adult patients with moderate to severe AD, 30.4% had low BMD (defined by a Z-score of ≤  − 1), with more men and being independent of the cumulative dose of topical and oral corticosteroids during the 5 years prior to inclusion^5^. They only found a trend toward an increased risk of low BMD with higher use of topical and oral corticosteroids with logistic regression^5^. In a comparative study of 43 children with severe eczema who were using topical corticosteroids and 73 healthy children, children with severe eczema had lower lumbar spine BMD. Among 43 children with severe eczema, six were also taking oral cyclosporine and they had lower lumbar spine BMD compared with those only on topical corticosteroids, suggesting that low BMD was primarily mediated by oral cyclosporine use rather than by topical corticosteroids^21^. Cyclosporine is a potent immunomodulatory drug that is very effective in treating severe recalcitrant eczema. Although the clinical benefits of oral cyclosporine, it is associated with several side effects, notably renal impairment, hepatic dysfunction, hypertension, and many investigators believe that cyclosporine may induce severe changes in bone metabolism^21^. An increasing evidence indicates that cyclosporine causes bone loss in humans, and in rats, high-dose cyclosporine use produces a high-turnover osteopenia with decreased bone mass, and these deleterious effects are more susceptible to trabecular bone than cortical bone^21^.

Many epidemiologic studies have demonstrated an association between osteoporosis and AD. According to a nationwide population-based study from Taiwan, when AD patients aged 20–49 years were compared with 1:1 age-gender matched controls without AD, 1.02% of AD patients and 0.36% of non-AD controls developed osteoporosis during the follow-up period^4^. The risk of osteoporosis was associated with female sex, older age, advanced Charson comorbidity index, depression, and systemic corticosteroid use within 5 years^4^. This population is in common with our study in the age of population included as 20–49, but the AD population in this study had significantly higher comorbidities including hypertension, diabetes, chronic kidney disease, and so on, as well as significantly higher rates of systemic corticosteroid use compared to non-AD matched controls. Hence, we assume that this could attribute the bone mass of the AD group and affect the increased rates of developing osteoporosis in the AD population in that study, which is different from our study of an AD population who did not have a significant increase in those comorbidities except for asthma. In a US study assessing the association of osteopenia and osteoporosis (using disease codes) in adults aged ≥ 50 years with physician-diagnosed AD from using the largest all-payer emergency department and inpatient database, AD was associated with increased odds of 1.31 (95% CI 1.12–1.54) for osteoporosis and 1.25 (95% CI 1.24–1.26) for osteopenia, suggesting that AD patients aged ≥ 50 years might benefit from increased screening for osteoporosis and osteopenia^26^.

Furthermore, increased fracture risk and injury have been reported in adult patients with AD in large population studies. In a matched cohort study from the UK including adults aged ≥ 18 years with atopic eczema and matched (age, sex, and general practice) controls without eczema, the authors compared the risk of fractures between the two groups^6^. In a comparison of 526,808 people with atopic eczema and 2,569,030 people without atopic eczema, those with eczema had an increased risk of hip, pelvic, spinal, and wrist fractures^6^. Fracture risk increased with increasing eczema severity, and the strongest associations were found in people with severe eczema for the spinal, pelvic, and hip^6^. In a prospective questionnaire-based population study from the US, adults with eczema had an increased risk of fracture and bone or joint injury (adjusted OR 1.67; 95% CI 1.21–2.33), and it increased gradually with age, peaking at 50–69 years and decreasing thereafter. Fracture risk increased with eczema severity and the associations persisted after adjusting oral glucocorticoids use^6^.

These inconsistencies between studies may be due to differences in study design, study population, sample size, a cut-off value of Z-score, the definition of osteoporosis, history of AD treatments (topical steroids [mild, moderate, high potency], topical tacrolimus, oral/systemic steroids, oral cyclosporine dose and duration, etc.), comorbidities of study population, and follow-up periods. Further studies are required to confirm these findings, determine their major risk factors, determine the effects of AD treatments, and find optimal strategies to reduce these comorbidities.

Among AD patients, early age at diagnosis of AD, longer duration of AD, lower BMI, rural living (for men), less educated, low vitamin D level, late menarche, and higher number of pregnancies (for women) were associated with low BMD. Low vitamin D levels were associated in women with AD, while an inverse association was found in men with AD, indicating that vitamin D deficiency had greater effects in women than in men. The late age of menarche and a higher number of pregnancies were associated with low bone mass among women with AD. An Association has been reported between late age at menarche and increased risk of osteoporosis/osteoporotic fractures explain the findings due to the shorter exposure period to circulating estrogen, which is a vital hormone for bone formation^6,27–29^. On the other hand, during pregnancy, bone absorption is increased^30^. Taken together, late menarche age and higher pregnancy numbers in AD patients might also affect bone mass towards bone absorption, leading to low BMD. Notably, we found that early onset and longer duration of AD were associated with low BMD. Chronic underlying inflammatory processes or as an indirect representation of severe disease activity, these early onset and longer duration of AD could be important history of AD patients when estimating bone health and osteoporosis risk. By using this information about demographics, AD history, and obstetric history of women, physicians can offer tailored counseling for bone health in adult patients with AD.

Nevertheless, several limitations of this study need to be mentioned. First, the KNHANES is a retrospective cross-sectional study, which limits the interpretation of causality between AD and osteoporosis. In addition, there were no long-term follow-up data on future osteoporosis and fracture risk in this young patient with AD. Therefore, a more careful interpretation is required to reach a definite conclusion. Second, the AD diagnosis was based on a self-reported questionnaire; additionally, a previous AD diagnosis was performed by a doctor of various specialties (general physician, dermatologist, pediatrician, etc.). These limitations may attenuate the accuracy of the AD diagnosis. Another major limitation of this study was the insufficient information on the time point and the duration of AD treatment and the type of AD treatment (topical steroids, topical tacrolimus, systemic steroids, cyclosporine, etc.) as well as AD severity, which may affect BMD of patients with AD. Additionally, as AD patients were identified based on the positive response to the self-reported questionnaire if a patient had ever been diagnosed as AD in one’s lifetime, there is a possibility that AD patients with complete remission may also be included. Lastly, we did not include biochemical information, such as calcium, phosphorus, parathyroid hormone, and thyroid function tests. This limitation may lead to the potential pitfall of passing over other secondary causes of osteoporosis in the young population. However, this study has strength by performing propensity score weighting not only for age and sex, but also for vitamin D level, alcohol consumption, and smoking habit, which might affect BMD and z-score. Furthermore, this study has importance since we focused on young adults population who are socially and physically active in our society and because those who have relatively have less burden of comorbidities (i.e., metabolic diseases) and not suffering from the abrupt hormonal change (i.e. postmenopausal) that have an enormous effect on bone, we can estimate the direct interaction of bone health among AD patients.

In conclusion, the prevalence of low BMD defined by Z-scores was not significantly different between the young patients with AD and the age/sex-matched control group. Although the increased risks of osteoporosis and fracture in young patients with AD still need to be established, our study using a large and meticulous case–control propensity score weighting design provides evidence on comparable BMD between young adult patients with AD and non-AD controls. Further long-term and large-scale studies are needed to determine the bone health and related risk factors in AD with well-stratified grouping of patients with AD.

## Supplementary Information

Supplementary Table 1.

## Data Availability

The dataset generated during the current study is available upon reasonable request from the corresponding author, Kyeong Jin Kim, (jins0707@korea.ac.kr).

## Code availability

The code generated during the current study is available upon reasonable request from the corresponding author, Kyeong Jin Kim, (jins0707@korea.ac.kr).
