# Supplementary material for: Bone mineral density and osteoporosis risk in young adults with atopic dermatitis
Source: Sci Rep. 2021 Dec 20;11:24228. doi: 10.1038/s41598-021-03630-z (PMC8688494; doi:10.1038/s41598-021-03630-z)
Supplement: Supplementary file 1 — Supplementary Table 1. [file 41598_2021_3630_MOESM1_ESM.docx]

Supplemental Data

**Supplementary Table 1.** Weighted mean BMD references for age and sex

|  |  | **Total hip (mg/cm^2^)** | |  | **Femoral neck (mg/cm^2^)** | |  | **Lumbar spine (mg/cm^2^)** | |
| --- | --- | --- | --- | --- | --- | --- | --- | --- | --- |
|  | N | Mean | SD |  | Mean | SD |  | Mean | SD |
| Total (male) | 11,585,713 | 998.2 | 120.9 |  | 862.0 | 128.5 |  | 987.3 | 123.7 |
| Age groups (years) |  |  |  |  |  |  |  |  |  |
| 19–24 | 1,770,546 | 1,043.0 | 122.4 |  | 940.1 | 129.1 |  | 1,008.8 | 119.4 |
| 25–29 | 1,863,862 | 1,000.6 | 124.2 |  | 891.2 | 134.7 |  | 994.9 | 121.9 |
| 30–34 | 1,886,346 | 994.1 | 117.2 |  | 865.1 | 120.1 |  | 994.8 | 118.3 |
| 35–39 | 2,064,772 | 984.9 | 118.9 |  | 840.3 | 118.3 |  | 980.0 | 121.1 |
| 40–44 | 1,984,955 | 988.2 | 116.9 |  | 832.0 | 114.6 |  | 979.8 | 120.8 |
| 45–49 | 2,015,232 | 984.0 | 116.5 |  | 814.9 | 113.9 |  | 969.8 | 134.8 |
| Total (female) | 10,111,944 | 898.0 | 107.0 |  | 765.0 | 103.8 |  | 984.5 | 115.8 |
| Age groups (years) |  |  |  |  |  |  |  |  |  |
| 19–24 | 513,127 | 902.7 | 97.3 |  | 798.3 | 103.4 |  | 948.7 | 103.5 |
| 25–29 | 2,739,870 | 885.2 | 103.3 |  | 770.9 | 103.0 |  | 963.7 | 107.4 |
| 30–34 | 1,607,625 | 886.4 | 109.7 |  | 759.8 | 103.6 |  | 990.3 | 113.4 |
| 35–39 | 1,934,415 | 902.6 | 105.8 |  | 761.7 | 104.3 |  | 1,000.3 | 116.3 |
| 40–44 | 1,811,540 | 912.1 | 108.4 |  | 763.0 | 102.6 |  | 1,000.2 | 116.7 |
| 45–49 | 1,505,368 | 908.9 | 109.9 |  | 754.7 | 103.7 |  | 988.5 | 126.7 |
